# Virtual harm reduction efforts for Internet gambling: effects of deposit limits on actual Internet sports gambling behavior

**DOI:** 10.1186/1477-7517-5-27

**Published:** 2008-08-06

**Authors:** Anja Broda, Debi A LaPlante, Sarah E Nelson, Richard A LaBrie, Leslie B Bosworth, Howard J Shaffer

**Affiliations:** 1 Coordination Center for Clinical Trials Leipzig (KKSL), Germany; 2 Division on Addictions, Cambridge Health Alliance, USA; 3 Harvard Medical School, USA

## Abstract

**Background:**

In an attempt to reduce harm related to gambling problems, an Internet sports betting service provider, ***bwin ***Interactive Entertainment, AG (***bwin***), imposes limits on the amount of money that users can deposit into their online gambling accounts. We examined the effects of these limits on gambling behavior.

**Methods:**

We compared (1) gambling behavior of those who exceeded deposit limits with those who did not, and (2) gambling behavior before and after exceeding deposit limits. We analyzed 2 years of the actual sports gambling behavior records of 47000 subscribers to ***bwin***.

**Results:**

Only 160 (0.3%) exceeded deposit limits at least once. Gamblers who exceeded deposit limits evidenced higher average number of bets per active betting day and higher average size of bets than gamblers who did not exceed deposit limits. Comparing the gambling behavior before and after exceeding deposit limits revealed slightly more unfavorable gambling behavior after exceeding deposit limits.

**Conclusion:**

Our findings indicate that Internet gamblers who exceed deposit limits constitute a group of bettors willing to take high risks; yet, surprisingly, they appear to do this rather successfully because their percentage of losses is lower than others in the sample. However, some of these gamblers exhibit some poor outcomes. Deposit limits might be necessary harm reduction measures to prevent the loss of extremely large amounts of money and cases of bankruptcy. We discuss how these limits might be modified based on our findings.

## Background

The Internet is a relatively new medium available for wagering. Research indicating how many people participate in Internet gambling is scarce. Two empirical studies published prevalence estimates of Internet gambling among the US general population: these studies reported rates of 0.3% [1] and 4% [2]. Among 1294 adults from a representative sample in Ontario, 5.3% reported having gambled on the Internet during the past 12 months [3]. Using a representative national sample from the United States, researchers reported a lower rate of 2.5% for college students [4]. Although some observers note that Internet gambling growth is slow compared to other forms of gambling, e.g. casinos and lottery [5], Internet gambling is prolific and growing [6]. Therefore, examining the influence of Internet gambling on public health is important.

Research examining land-based gambling suggests that adverse gambling-related outcomes often include financial distress, emotional and physical deterioration, and damaged interpersonal relationships [7]. Some research suggests that disordered gambling relates to poor mental health, such as personality and psychiatric disorders [8,9]. Researchers, public policy makers, and public health officials have argued that Internet gambling is associated with similar public health threats [10-13]. One study reported that Internet gambling was linked to pathological gambling and associated with poor physical and mental health [14]. Because this is the only study providing empirical data about health correlates of Internet gambling, and because this study provides results based on retrospective self-reports of a locally restricted sample of patients in clinic waiting areas, what we actually know about the health dangers of Internet gambling remains limited.

Speculations about potential hazards particular to Internet gambling include the apparent lack of fail-safes, such as the inability to protect individuals who are underage or people known to have gambling-related problems and to prevent gambling while intoxicated or gambling at work [15]. However, the Internet also provides a unique opportunity for implementing special safeguards and harm reduction efforts. For example, tracking software can record all gambling online activity, which companies could potentially use to control the extent of gambling by specific users. Web-based technology could limit the time per gambling session or the amount of money participants can use to gamble. Recent recommendations for Internet gambling operators include accepting payments with credit cards only, providing options to self-limit gambling expenditure, and providing options that allow users to self-exclude from an Internet site [16].

In this study, we explore a harm reduction feature currently unique to Internet gambling. As part of their corporate social responsibility agenda, a large Internet sports betting service provider, ***bwin ***Interactive Entertainment, AG (***bwin***), imposes limits on the amount of money that users can deposit into their online gambling accounts within a given time period. When a user tries to deposit more than the allowed amount, ***bwin ***sends the user a notification message about the attempt to exceed deposit limits and rejects the attempted deposit. We expected that users who received a notification message constitute a group of extremely engaged gamblers, and we therefore hypothesized that exceeding deposit limits would be associated with unfavorable gambling behavior, such as excessively large betting, high losses or high frequency of playing (i.e., high financial and/or temporal engagement). Furthermore, we expected that receiving a notification message would act as a warning sign to users; consequently, we hypothesized that exceeding deposit limits would attenuate gambling behavior that followed exceeding the limit. To examine these possibilities, this study compares (1) the gambling behavior of those who exceeded deposit limits with those who did not, and (2) the gambling behavior of consumers before and after exceeding deposit limits.

## Methods

### Sample

The research cohort included 48114 people who registered with ***bwin ***between February 1 and February 28, 2005, and who deposited money in their accounts before February 28, 2007. ***bwin ***is primarily an Internet sports gambling service, offering two types of sports bets: fixed-odds bets and live-action bets. Fixed-odds bets are made on the outcomes of sporting events or games before the events begin. The amount paid for a winning bet is set (fixed) by the betting service at the time of the bet. Live-action bets are made while the event is in progress. In addition to bets on the outcome of the event, the betting service offers bets on selected outcomes within the sporting event (e.g., which side will have the next corner kick). Fixed-odds bets are relatively slow-cycling betting propositions. The outcomes of a bet are generally not known for hours or days later. In contrast, live-action bets provide relatively quick-paced betting propositions posed in real-time during the progress of a sporting event.

Some subscribers in the cohort did not engage in fixed-odds or live-action sports gambling (n = 1114, < 3%). Consequently, these subscribers were excluded from the study, leaving 47000 sports-betting subscribers for the current analysis. This cohort consisted of 43222 (92.0%) men and 3778 (8.0%) women. The mean age of subscribers was 30.3 years (SD = 9.9) and the cohort included people from 84 countries, with most people (n = 26955, 57.4%) from Germany, followed by Turkey (n = 2846, 6.1%), Poland (n = 2834, 6.0%), Spain (n = 2754, 5.9%), and Greece (n = 2586, 5.5%). The majority, 31544 (67.1%), placed both fixed-odds and live-action bets, 14723 (31.3%) played fixed-odds only, and 733 (1.6%) played live-action only.

### Measures

***bwin ***prepared a dataset of the actual Internet sports gambling behavior of this cohort for the 2+-year period, between February 1, 2005 and February 28, 2007. More specifically, this dataset included the daily aggregates of betting activity (i.e., the aggregate number of bets, amount of money wagered, and amount of money won for fixed-odds and live-action sports betting per calendar day) for all participants in the cohort.

#### Exceeding Deposit Limits

***bwin ***provides several ways of limiting the amount of money that users can deposit in their accounts. By default, ***bwin ***does not allow users to deposit more than 1000 Euros per 24 hours or 5000 Euros per 30 days (or currency equivalents). One exception to this default is a flexible limit system, which automatically increases allowable deposit limits by the subscribers' amount of winnings from gambling. A second exception occurs when subscribers can evidence exceptional financial means. In such cases, users may have higher deposit limits. At the other end of the spectrum, users can choose to set for themselves lower maximum deposit amounts per 30 days. Users repeatedly can adjust these self-limits to their needs.

Exceeding any deposit limit leads ***bwin ***to issue a notification message. Although we have information about if and when a user received such notification, we do not have information about the type of limit (i.e., self or company) that initiated the notification message. Thus, this study explores the combined effects of exceeding company- and self-imposed deposit limits.

#### Gambling Behavior

Based on the daily aggregates of betting activity, we computed four measures of gambling behavior for each user: percentage of days within the active period from first to last betting day on which the user placed bets (i.e., percent active betting days); the average number of bets per active betting day; the average size of bets in Euros; and a categorical measure of percent lost. These measures are more adequate than gross totals of number of bets or money wagered when comparing the gambling behavior of different users. Each measure was computed for fixed-odds and live-action betting separately, aggregated across the total 2-year observation period. Further, within the subset of people who received a notification message, each measure was computed for the period of time before as well as after the first receipt of a notification message (note that the day of receipt of the notification message was defined as an 'after' day).

We defined the percentage of active betting days as the percent of days within the interval from the first to the last betting day that included a bet. We obtained the average number of bets per active betting day by dividing the total number of bets made by the total number of active betting days, and the average size of bets in Euro by dividing the total money wagered by the total number of bets. These two gambling behavior measures were highly positively skewed with many cases on the left and fewer cases (but still substantial numbers due to the large sample size) on the right side of the distribution. Log-transformations were performed to generate normal distributions for these measures.

We calculated the percentage of losses by subtracting the total amount of winnings from the total amount of wagers and dividing the difference by the total amount of wagers. This measure was highly negatively skewed and transformations did not help approximate a normal distribution. We therefore categorized this variable to capture (1) users who were overall winners (negative percentage of losses), (2) users with the lowest percentage of losses (operationally defined as losses of 0 to <20%), (3) users with an intermediate percentage of losses (i.e., 20 to <80%), and (4) users with the highest percentage of losses (i.e., 80 to 100%). We chose the cut-point of the lowest percentage of losses to approximately agree with the expected losses, which according to the target returns expected by the operator are approximately 13% for fixed-odds betting and 6% for live-action betting. The 20% cut-point reflects the nearest rounded percentage. For the cut-point of the highest percentage of losses, we applied the same 20% margin, and the remaining percentage of losses was categorized as intermediate.

#### Most Involved Bettors

We defined *most involved bettors *(MIB) subgroups as the top 1% of the sample regarding the total number of bets, total amount of wagers, and net loss (i.e., subtracting the total amount of winnings from the total amount of wagers) on fixed-odds or live-action betting. We used a scree-type analysis of centile plots to empirically identify these 1%-subgroups [17]. This strategy allowed us to classify MIB into six non-exclusive groups: (1) total number of bets; (2) total amount of wagers; (3) net loss for fixed-odds players (each of these groups n = 462); (4) total number of bets; (5) total amount of wagers; and (6) net loss for live-action players (each of these group n = 322). A total of 984 users belonged to at least one of the fixed-odds MIB groups (2.13% of 46267 fixed-odds players), and a total of 613 users belonged to at least one of the live-action MIB groups (1.90% of 32277 live-action players).

### Analyses

In addition to providing descriptive statistics, we conducted two primary comparative analyses. First, we examined differences in gambling behavior between users who did and did not exceed deposit limits using independent-samples tests. These tests employed the gambling behavior measures that were aggregated across 2 years. We also looked to see whether the proportion of most involved betters was greater among individuals who exceed limits compared to those who did not. Second, we analyzed individuals' differences in gambling behavior before and after exceeding deposit limits within users who exceeded limits using paired-samples tests. These tests compared the gambling behavior measures that were created for the period of time before and after receipt of the notification message.

The procedures of limiting deposits and sending notifications were only in effect starting late September 2005, about 8 months after the beginning of our study. Users of our cohort experienced no restrictions to the amount of money they could deposit during the first 8 months after they registered with ***bwin***. This could potentially bias our findings: On one side, only a subset of people who registered in February 2005 was still active in September 2005 (e.g., of the 47000 sports, 27726 or 59% had deposited money after September 2005) and thus could experience the new deposit limit policies. Short-term bettors might exhibit a low extent of gambling behavior, potentially resulting in overestimating the differences between users who did and did not exceed limits. We therefore repeated the analyses within the subset of users who had deposited money after September 2005.

Alternatively, certain deposit activities could have been possible before September 2005, but might have conflicted with company-imposed or self-imposed deposit limits after September 2005. For example, people could deposit very large amounts during the first 8 months but not after September 2005. People might have chosen to self-limit their deposit amount but had no option to do so before September 2005. We had no means of identifying people who would have been subject to one of the limits before September 2005, and thus no means of excluding these people from the analyses. However, in our analyses these people are considered users who did not exceed limits, yielding conservative estimates for the comparisons of users who did and did not exceed limits.

Analyses involving the average number of bets per active betting day and the average size of bets in Euro used the log-transformed variables; however, we report means, standard deviations, and medians for the untransformed variables for descriptive purposes.

## Results

### Descriptive Statistics

Of the 47000 sports bettors, 160 (0.3%) had received at least one notification message about exceeding deposit limits. Five (3.1%) were women and 155 (96.9%) were men, and the mean age was 30.8 years (SD = 9.2). Most of the bettors who exceeded limits played both types of games: 159 (99.4%) were fixed-odds players and 149 (93.1%) were live-action players; 148 of the 149 who played live-action also played fixed-odds. Among users who placed both fixed-odds and live-action bets, 0.5% (n = 148) received a notification message, compared to 0.1% (n = 11) of users who played fixed-odds only and 0.1% (n = 1) of users who played live-action only (χ^2 ^= 46.95, df = 2, p < .001).

These 160 notified users received between 1 and 267 notification messages, with a mean of 14 messages (SD = 29, Median = 6). Of the 160 users, 5 (3.1%) stopped depositing money in their accounts after receiving the notification message. One user had tried to deposit more than the allowed amount with the very first deposit. The mean number of deposits before receiving the notification message was 57 (SD = 89, Median = 20) with a range of 1 to 796. The mean number of days between the date of the first deposit and the date of the first notification message was 372 (SD = 184, Median = 380) with a range of 0 to 741.

To describe the general distribution of deposits, we examined the maximum amount deposited per 24-hour and 30-day period among the 46840 sports bettors who never received a notification message. Table 1 reports the mean (SD) and centiles for this measure. The vast majority of users never came close to the limits of 1000 Euros/24 hours or 5000 Euros/30 days.

**Table 1:** Descriptive statistics for maximum amount of euros deposited by time period

	Euro in 24 hours	Euro in 30 days
Mean (SD)	111 (258)	243 (725)
Percentile		
25^th^	25	30
50^th ^(Median)	40	50
75^th^	100	150
90^th^	250	500
95^th^	500	1050
99^th^	1000	3768

### Comparing Gambling Behavior between Users who Did and Did Not Exceed Deposit Limits

Table 2 presents a comparison of gambling behavior aggregated across 2 years for users who did and did not exceed their established deposit limits. Results were similar for fixed-odds and live-action betting. The percentage of active betting days for these groups was not significantly different. The average number of bets per active betting day and the average size of bets were higher among users who exceeded deposit limits compared to users who did not exceed deposit limits. The distribution of the categorized percentage of losses was more favorable among users who exceeded deposit limits; that is, the likelihood of the lowest percentages of losses was significantly higher and the likelihood of the intermediate and the highest percentages of losses were significantly lower among users who exceeded deposit limits.

**Table 2:** Gambling behavior in users who did and did not exceed deposit limits

		Fixed-odds (n = 46267)			Live-action (n = 32277)		
					
Gambling behavior measure		Users who did exceed limits (n = 159)	Users who did not exceed limits (n = 46108)	Difference test	Users who did exceed limits (n = 149)	Users who did not exceed limits (n = 32128)	Difference test
Percentage of active betting days	Mean (SD)	21 (20)	25 (29)		26 (28)	31 (37)	
	Median	14	13		16	11	
	Log Mean (SD)	--	--	t = -1.90	--	--	t = -1.71
Average number of bets per active betting day	Mean (SD)	7 (13)	4 (7)		8 (14)	4 (5)	
	Median	3	2		4	3	
	Log Mean (SD)	0.60 (0.40)	0.44 (0.32)	t = 6.12*	0.68 (0.42)	0.46 (0.34)	t = 7.98*
Average size of bet in Euro	Mean (SD)	25 (55)	11 (30)		27 (41)	11 (25)	
	Median	8	4		12	4	
	Log Mean (SD)	0.96 (0.60)	0.67 (0.51)	t = 7.14*	1.07 (0.58)	0.65 (0.53)	t = 9.59*
Categorized percentage of losses							
Overall winners	n (%)	26 (16.4)	6755 (14.7)		25 (16.8)	6924 (21.6)	
Lowest percentage of losses	n (%)	59 (37.1)	12367 (28.8)		73 (49.0)	10548 (32.8)	
Intermediate percentage of losses	n (%)	56 (35.2)	19338 (41.9)		40 (26.8)	9533 (29.7)	
Highest percentage of losses	n (%)	18 (11.3)	7648 (16.6)	Chi^2 ^= 10.91*	11 (7.4)	5123 (15.9)	Chi^2 ^= 20.58*

* p < .05.

Despite losing a smaller proportion of what they wagered, users who exceeded limits still, on average, lost significantly more than users who did not exceed limits. That is, the mean net loss on fixed-odds of users who did exceed limits was 1,135 Euro (SD = 2,766, Median = 213) compared to 185 Euro (SD = 1,028, Median = 50) for users who did not exceed limits (t = 11.51, p < .001) (live-action: 1,975 Euro, SD = 5,569, Median = 135, compared to 187 Euro, SD = 1,414, Median = 13, t = 14.91, p < .001).

Exceeding deposit limits was a significant predictor of being in the MIB subgroups. For example, 14.5% of users who received notification messages, belonged to at least one of the fixed-odds MIB groups, compared to 2.1% who did not; this association yielded an odds ratio of 7.95 (95% CI 5.08 – 12.42). Further, 14.8% of users who received notification messages compared to 1.8% of those who did not, belonged to at least one of the live-action MIB groups; this association yielded an odds ratio of 9.24 (95% CI 5.84 – 14.64). Table 3 presents the associations between individual MIB groups and exceeding limits.

**Table 3:** Proportions of most involved bettors (MIB)^1 ^among users who did and did not exceed deposit limits

	Fixed-odds (n = 46267)			Live-action (n = 32277)		
MIB group	Users who did exceed limits (n = 159)	Users who did not exceed limits (n = 46108)	Odds ratio (95% CI)	Users who did exceed limits (n = 149)	Users who did not exceed limits (n = 32128)	Odds ratio (95% CI)

Total number of bets	6.3%	1.0%	6.78 (3.55 – 12.95)	6.0%	1.0%	6.53 (3.30 – 12.94)
Total amount of wagers	8.8%	1.0%	9.84 (5.64 – 17.17)	11.4%	0.9%	13.44 (8.01 – 22.55)
Net loss	9.4%	1.0%	10.64 (6.20 – 18.26)	12.1%	0.9%	14.38 (8.68 – 23.85)

^1^MIB are defined as the top 1% of the sample regarding the total number of bets, total amount of wagers, and net loss on fixed-odds or live-action betting.

Within the group of people who exceeded limits, we compared users who belonged to at least one of the fixed-odds or live-action MIB groups to users who did not belong to a MIB group on the gambling behavior measures. Users belonging to a MIB group had a higher percentage of active betting days and a higher average number of bets per active betting day on fixed-odds and live-action, and a higher average size of bet on live-action. The distribution of the categorized percentage of losses was not significantly different between users who did and did not belong to a MIB group.

### Comparing Gambling Behavior Before and After Exceeding Deposit Limits

Table 4 shows the comparison of gambling behavior before and after exceeding deposit limits. Of the 159 fixed-odds players, 143 had activity both before and after exceeding deposit limits and are included in Table 4; likewise, of the 149 live-action players, 105 had activity both before and after and are included in the Table.

**Table 4:** Gambling behavior before and after exceeding deposit limits

		Fixed-odds (n = 143)			Live-action (n = 105)		
					
Gambling behavior measure		Before	After	Difference test	Before	After	Difference test
Percentage of active betting days	Mean (SD)	23 (22)	26 (26)		27 (23)	26 (25)	
	Median	14	17		20	19	
	Log Mean (SD)	--	--	t = -1.37	--	--	t = 0.11
Average number of bets per active betting day	Mean (SD)	6 (12)	7 (15)		11 (18)	9 (17)	
	Median	3	3		6	5	
	Log Mean (SD)	0.59 (0.38)	0.49 (0.43)	t = 4.10*	0.78 (0.42)	0.71 (0.44)	t = 2.47*
Average size of bet in Euro	Mean (SD)	21 (48)	44 (107)		25 (43)	32 (45)	
	Median	7	9		11	17	
	Log Mean (SD)	0.90 (0.57)	1.04 (0.70)	t = -3.63*	1.03 (0.57)	1.17 (0.59)	t = -4.27*
Categorized percentage of losses							
Overall winners	n (%)	30 (21.0)	20 (14.0)		17 (16.2)	21 (20.0)	
Lowest percentage of losses	n (%)	41 (28.7)	40 (28.0)		59 (56.2)	43 (410)	
Intermediate percentage of losses	n (%)	56 (39.2)	47 (32.9)		24 (22.9)	31 (29.5)	
Highest percentage of losses	n (%)	16 (11.2)	36 (25.2)	Chi^2 ^= 15.30	5 (4.8)	10 (9.5)	Chi^2 ^= 9.38

* p < .05.

Again, similar patterns of results emerged for fixed-odds and live-action betting. The percentage of active betting days and the distribution of the categorized percentage of losses did not change. The average size of bet increased and the average number of bets per active betting day decreased after exceeding deposit limits.

### Analyses for Users Who Deposited Money after September 2005

To control for potential biases that might result from the notification messages being introduced only after September 2005, we repeated all the above analyses with the subset of 27726 users (59% of the total sample) who had still deposited money after September 2005. These analyses compare the 159 fixed-odds players who exceeded limits with 27442 users who did not exceed limits, and the 149 live-action players who exceeded limits with 21433 users who did not exceed limits. Overall these analyses yielded the same pattern of results, although the percentage of active betting days was significantly different between the limit-exceeding and non-limit-exceeding groups for fixed-odds betting and the distribution of the categorized percentage of losses was not significantly different between the two groups for either fixed-odds or live-action betting. The odds ratios for belonging to the MIB subgroups were slightly lower overall: between 4.06 and 6.53 for the fixed-odds MIB groups and between 4.37 and 9.78 for the live-action MIB groups. The analyses of gambling behavior before and after exceeding deposit limits necessarily are identical to the overall results.

## Discussion

The company-imposed or self-imposed deposit limits affected only a minority of ***bwin ***Internet sports bettors. Very few people, only 0.3% of our sample, ever tried to exceed these deposit limits. Furthermore, the vast majority of the sample (i.e., 95%) never deposited more than 500 Euro per 24 hours, half the maximum allowed 1000 Euros, and never deposited more than 1050 Euro per 30 days, a fifth of the maximum allowed 5000 Euros. This means that ***bwin ***could reduce the deposit limits substantially (e.g., by half) and still most people would not exceed these limits.

One reason for the finding that the deposit limits were hardly exceeded might be that the sports bettors are highly responsible gamblers who bet for fun and spend relatively low amounts on betting. Another reason might be that users are well aware of ***bwin's ***deposit limits policies and purposely avoid violating them. The deposit limits are presented as part of the general terms and conditions that every user needs to accept when opening an account with ***bwin***. Our findings seem to indicate that knowing about the deposit limits prevented some bettors from exceeding the deposit limits and subsequently from losing money. If this is correct, then the mere provision of deposit limits can serve as a harm reduction device.

We examined whether the deposit limits seemed to safeguard the gambling behavior of the minority that exceeded the deposit limits. People who exceed deposit limits constitute a group of bettors who are willing to place larger bets than people who do not exceed deposit limits; yet, they appear to do this in a manner that keeps their percentage of losses lower than others in the sample. Although the percentage of losses might be more favorable among people who exceed limits, compared to people who do not exceed limits, their net loss still is significantly higher. Because these bettors place very large bets they are at high risk for losing very large amounts of money.

We identified exceeding limits as a strong predictor for being in the MIB subgroups. People who exceed limits are about 6 to 14 times more likely to belong to the various MIB groups. Thus, exceeding the limits is associated with a high likelihood of being in the group of bettors that bet, wagered and/or lost the most; these activities are possible indicators of disordered gambling. Consistent with this notion, we found that among people who exceed limits, people who belong to MIB groups show more intensive gambling behavior than people who do not belong to MIB groups. That is, those who belong to MIB groups bet more often, place more bets, and place larger bets.

Our comparison of the gambling behavior before and after exceeding limits found that exceeding the limits did not have a diminishing effect on gambling behavior. The number of bets was the only measure of gambling behavior that evidenced a minor decrease after exceeding limits. This decrease was offset by a steep increase in the size of bets after exceeding limits. The number of days of play and the percentage of losses did not change. Thus, we found no indication that receiving the limit notification message influences users to curtail their betting activity. Rather, the findings suggest that exceeding deposit limits encourages players to shift their strategy; they begin to make more calculated, informed risks with single large bets compared to before exceeding the deposit limit.

The finding that the feedback about a violation of a policy or regulation does not have the intended harm-reducing effect is a finding consistent with other evidence about regulating behavior. For example, people who were given feedback that their blood alcohol levels exceeded legal drink-drive limits have been nonetheless subsequently observed to drive [18-20]. Drivers who received speeding tickets have been shown to be at increased risk of receiving subsequent speeding tickets [21]. Likewise, smokers who were given biomedical feedback indicating negative effects of smoking did not initiate appreciable changes towards quitting smoking [22].

No differences emerged in the patterns of results for fixed-odds and live-action betting. Fixed-odds and live-action propositions might differ in the extent of skill required to place successful (i.e., winning) bets. Whereas placing a successful bet in fixed-odds might be determined more by skill (or knowledge) than by chance, placing a successful bet in live-action likely is determined more by chance than by skill. Thus, we could have expected our findings to mirror the differing outcomes of games of skill versus games of chance. Our findings instead show that, with regard to evaluating the risk of disordered gambling among people who exceed deposit limits, distinguishing fixed-odds from live-action betting does not provide additional information.

### Limitations

Our study examines conceptually different deposit limits. Some limits are mandatory and imposed by ***bwin***: the default limits of 1000 Euros per 24 hours or 5000 Euros per 30 days, and the increase of the default limits by the amount of winnings. The users voluntarily impose other limits: restrictions to a lower amount than the default limits, or exemption from default limits for users with exceptional financial means. When exploring the effects of exceeding deposit limits, we combined the different deposit limits. Although different deposit limits are examined in this study, an essential similarity of all deposit limits is that they represent specific, predetermined maximum values that certain users are not willing to or are not able to comply with. To this extend, the current study investigates effects of exceeding pre-set deposit limits.

We can posit that different types of limits might be associated with different effects on gambling behavior. Therefore, an analysis differentiating the types of limits would have been desirable. Unfortunately, no information was available about the type of limit that led to issuing a notification message; thus this analysis was not an option for this paper.

It is important to note that the procedures of limiting deposits and sending notification messages were not in effect during the entire two-year study period. We performed some statistical controls in our analyses to account for this fact, and the overall results remained largely unchanged. Thus, we consider our findings to reflect generalizable effects of deposit limits on Internet sports gambling behavior.

## Conclusion

The deposit limits examined in this study are part of the corporate social responsibility agenda of ***bwin***. This harm reduction practice is consistent with recommendations to integrate safety features for the prevention of disordered gambling into gambling websites [16].

This study indicates that current deposit limits affect only a very small minority of Internet sports bettors. The vast majority of Internet bettors seem to be able to regulate themselves and require little additional safeguards; however, some bettors can benefit from additional limits. Consequently, for Internet gambling operators reluctant to include harm reduction measures, an interesting message is that a company's financial loss due to imposing such safeguards such as deposit limits will be rather small and balanced by the promoting effect of being regarded as a socially responsible company.

In this study, we saw that the mandatory limits exceed what most people are willing to spend on Internet gambling activities. However, the mandatory limits also exceed what most people could possibly spend without taking substantial financial risks. Thus, while the current mandatory limits might help prevent the loss of extremely large amounts of money and cases of bankruptcy, these limits still allow users to transfer substantial amounts of money each day and each month, which can lead to financial problems for gamblers without sufficient financial means. For these cases, instead of the company-imposed limits, the self-imposed limits might have value.

This study shows that people who try to exceed deposit limits have some poor outcomes: a high likelihood of placing an extremely large number of bets, wagering extremely large amounts, and/or loosing extremely large amounts of money. These people constitute a group of bettors who appear to be willing to take high risks; yet, surprisingly, they appear to do this rather successfully because their percentage of losses (but not their net loss) is lower than others in the sample.

Without deposit limits, the behavioral and financial consequences of gambling might be even more adverse. These unintended consequences of Internet gambling indicate that the ***bwin ***deposit limits could aid in the prevention of adverse gambling-related consequences. More research is necessary to determine the extent of this influence and to monitor and revise such notification systems so that the promise of limits can be optimized. For example, recent research suggests that gambling activity, or behavioral engagement in gambling, might be as important to consider as financial considerations [23]. Such findings suggest that corporations need not limit harm reduction techniques to financially-related factors. Rather, techniques that account for temporally-related factors (e.g., amount of time spent gambling) remain open to consideration and examination. Online gambling companies would benefit from testing the harm reduction value of warning systems for amount of time spent gambling.

In this study, we examined deposit limits as a single harm reduction measure of a single Internet gambling provider. Unfortunately, users can sidestep such single safeguards easily; another Internet gambling provider is just a mouse-click away. To implement effective safeguards, concerted harm reduction efforts of companies, users, public health organs, and others are necessary. Ways of achieving this goal might include the development of policy requiring safety provisions as a prerequisite for licensing providers, or having companies cooperate to employ software programs and technology tools to regulate user gambling.

The findings of this study originate from actual gambling behavior and betting activity, without any direct contact with individual gamblers. A previous study analyzing Internet sports betting behavior in this manner indicated that, at the population level, gambling activity is moderate, as evidenced by analyses of time (e.g., people were active less than half the time possible, despite infinite access), activity (e.g., most placed less than 4 bet/day during such limited active periods), and expenditures (e.g., most placed bets less than 5 Euros) [4,17]. Future research needs to investigate how these findings compare with subjective assessments of perceptions and behaviors by the individual. If additional research supports the findings of this study, technology-based screening tools for gambling-related problems could incorporate the attempt to deposit more than the allowed amount of money as an early indicator of a person's vulnerability to disordered gambling.

## Competing interests

The authors declare that they have no competing interests.

## Authors' contributions

AB performed the statistical analyses and drafted the manuscript. RAL and HJS conceptualized the study and were instrumental in its design and coordination. DAL and SEN participated in the statistical analyses and were involved in drafting the manuscript. LBB made substantial contributions to the analysis and interpretation of the data. All authors read and approved the final manuscript.
